# Dysregulation of zinc finger protein, X-linked (ZFX) impairs cell proliferation and induces apoptosis in human oral squamous cell carcinorma

**DOI:** 10.1007/s13277-015-3292-7

**Published:** 2015-04-28

**Authors:** Hongzhi Ma, Fan Yang, Meng Lian, Ru Wang, Haizhou Wang, Ling Feng, Qian Shi, Jugao Fang

**Affiliations:** 1 Department of Otolaryngology-Head and Neck Surgery, Beijing Tongren Hospital, Capital Medical University, Beijing, 100730 China; 2 Key Laboratory of Otorhinolaryngology Head and Neck Surgery, Ministry of Education, Beijing Institute of Otorhinolaryngology, Beijing, 100005 China

**Keywords:** Oral squamous cell carcinorma, Zinc finger protein, X-linked, Proliferation, Cell cycle, Apoptosis

## Abstract

Zinc finger protein, X-linked (ZFX) is a transcriptional factor involved in many physiological processes such as embryonic stem cell survival and self-renewal. Though ZFX dysfunctions have been identified in variant human diseases and especially in cancers, its pathological roles have not been fully addressed. Here, we explored the relationship between ZFX expression and squamous cell carcinoma (SCC) of the tongue. We found that ZFX expression was significantly higher in tongue SCC tumors as compared to tumor-adjacent normal tissues. Furthermore, ZFX knockdown impeded cell proliferation, impaired colony formation ability, and lead to cell cycle arrest while induced cell apoptosis in human tongue squamous cell carcinoma cell line Tca-8113. Our results provide evidence suggesting that ZFX overexpression is associated with the development of tongue SCC and ZFX knockdown is a potential treatment for tumor suppression.

## Introduction

Tongue squamous cell carcinoma (SCC) is the most common malignant type in head and neck squamous cell carcinoma (HNSCC), which is among the top ten leading cancers worldwide [1]. Though remarkable progress has been achieved in clinical and basic research in the past three decades, the 5-year survival rate of tongue SCC patients showed little improvement and remained about 50 % [2]. Another major problem is that most tongue SCC cases showed lymph node metastasis at diagnosis, which indicated poor prognosis, and effective treatment are still underway at present. Though multiple factors including drinking and smoking have been implicated in the development and progression of tongue SCC [3], molecular signals and factors involved in tumorigenesis remain to be determined. Exploring novel molecules involved in tongue SCC will provide valuable insights for better diagnosis and treatment of human tongue SCC in future clinical studies.

Cys_2_His_2_ zinc finger (C2H2-ZF) proteins are the major class of DNA-binding proteins and have been implicated in diverse biological processes such as cell proliferation differentiation and cell survival while their dysfunctions have been linked to multiple human diseases including variant types of cancer [4]. Zinc finger protein, X-linked (ZFX) which is encoded by X-linked ZFX gene is a C2H2-ZF transcriptional factor composed of three functional domains including a DNA-binding domain containing 13 C2H2-type zinc fingers, an acidic transcriptional activation domain, and a nuclear localization sequence. It has been shown previously that ZFX is critical for the survival and self-renewal of embryonic and hematopoietic stem cells [5, 6]. In consistent with the role of C2H2-ZF proteins in cancer development, previous studies from our lab have shown that ZFX is aberrantly upregulated in human laryngeal squamous cell carcinoma and ZFX knockdown impaired cancer cell proliferation and apoptosis [7]. Furthermore, ZFX protein has been implicated in acute myeloid leukemia (AML) and acute T-lymphoblastic leukemia (T-ALL) [8]. However, the relationship between ZFX and tongue SCC remains poorly understood.

As both tongue SCC and laryngeal squamous cell carcinoma belong to HNSCC, here we examined the expression status of ZFX and unraveled significant expression differences between tumors and adjacent normal tissues derived from tongue SCC patients. In accord with previous studies showing that ZFX expression is increased in tumors, ZFX overexpression was observed in tongue SCC samples. Furthermore, ZFX expression in human tongue squamous cell carcinoma cell line Tca-8113 was inhibited efficiently by lentiviral-based small interference RNA (siRNA) strategy, and the impact of ZFX knockdown on cell proliferation, colony formation, cell cycle, and cell survival was investigated extensively, confirming the pathological role of ZFX for tongue SCC development and progression. So, our results provide confidential evidence that ZFX is quite a promising target for tongue SCC treatment.

## Materials and methods

### Patients, tumor samples, and immunohistochemistry (IHC) staining

This study was approved by the Research Ethics Board, and informed consent was obtained from all patients. A consecutive series of 30 tongue squamous cell carcinoma patients including 21 male and 9 female patients (between 24 and 85 years old with an average 59.7 years old) who underwent surgery between March 2011 and December 2013 was involved in this study. Patients could be classified according to tumor TNM stage system and shown in Table 1. None of the patients were treated with radiotherapy, chemotherapy, or other therapies before surgery. Specimens of tumor and of normal adjacent tissues were obtained, frozen in liquid nitrogen immediately, and stored at −80 °C for further analysis. ZFX immunohistochemical staining was performed as described previously [9]. Briefly, 4.0-μm-thick paraffin-embedded tissue sections were prepared and incubated with NBP1-80582 ZFX antibody purchased from Novus Company. Staining was done with an EliVision^TM^ plus kit (Fuzhou Maixin Biotech. Co., Ltd). Then, in each slice, five fields were selected randomly under high magnification (400×). The mean optical density (OD) of ZFX positive cells was counted in each field, and then, OD value of five fields was averaged for ZFX expression in each slice. OD value of each specimen was calculated as above, and paired *t* test was used to for OD value comparation between tumor and normal adjacent tissue samples. Leica dc300f microscope, Leica IM50 picture collecting system, and Leica Qwin software were used here.

**Table 1:** Patient information

Clinical pathological parameters	Case number
Gender	
Male	21
Female	9
TNM stage	
T1N0M0	4
T2N0M0	18
T2N2M0	1
T3N0M0	7

### Production of lentivirus expressing ZFX-siRNA

To inhibit ZFX expression, lentivirus expressing siRNA targeting ZFX specifically was produced as described previously [10]. Briefly, siRNA (siRNA sequence: gaGCCTGAGAATGATCATGGA) specifically targeting human ZFX gene (ZFX-siRNA) was designed while scrambled siRNA (scr-siRNA) with the following sequence was used as the negative control: TTCTCCGAACGTGTCACGT. To construct lentivirus expressing ZFX- or scr-siRNA, complementary single-strand DNA oligonucleotides were synthesized, annealed, and then inserted into lentiviral vector pGCSIL-GFP (GeneChem, Shanghai, China). Lentivector expression system (GeneChem, Shanghai, China) was selected to prepare desired lentivirus. Human tongue squamous cell carcinoma cell line Tca-8113 was selected and infected with lentivirus expressing ZFX- or scr-siRNA to determine the knockdown efficiency at RNA and protein level by real-time PCR and Western blot.

### Bromodeoxyuridine (BrdU) incorporation assay

Cell proliferation was assessed using BrdU incorporation assay. Human tongue squamous cell carcinoma cell line Tca-8113 was cultured and infected with lentivirus expressing ZFX or scrambled siRNA and incubated for another 48 h. Cells were then resuspended and seeded into 96-well plates at the proper density and cultured for another 1 or 4 days. Then, BrdU reagents were added with 1:100 dilution (10 μl/well). After 2–24 h, BrdU incorporation was analyzed using a BrdU Cell Proliferation ELISA kit (Cat. No. 11647229001, Roche Applied Science) following the manufacturer’s instructions. Briefly, cell fixation was performed using FixDenat (200 μl/well) for 30 min and blocked with 5–10 % BSA for another 30 min at room temperature. Then, cells were treated with diluted anti-BrdU-POD for 90 min at room temperature. Cells were incubated with substrate solution for 5–30 min in the dark after washing three times with washing buffer. After color development, the absorbance of the samples was measured at 450 nm to determine BrdU incorporation ratio.

#### MTT assay

Cell proliferation status was determined by MTT assay. Human tongue squamous cell carcinoma cell line Tca-8113 was infected with lentivirus expressing ZFX-siRNA or scrambled siRNA and incubated to reach logarithmic phase. Cells were then digested and resuspended thoroughly. Cell number was then counted using a hemocytometer, and 3000 cells per well were added in 96-well plates and cultured at 37 °C in a 5 % CO_2_ incubator. Cell proliferation was monitored for continuous 5 days with MTT assay. Each well was incubated with 20 μl of MTT solution (5 mg/ml) for 4 h. The culture medium was discarded, and 150 μl DMSO was added to dissolve formazan. 96-well plate was shaken for 5–10 min, and absorbance at 490/570 nm was examined with a microplate reader.

#### Cell apoptosis measurement

Cell apoptosis was detected using annexin V-APC apoptosis detection kit (eBioscience, 88–8007) and measured with flow cytometry. In brief, Tca-8113 cells were infected with lentivirus expressing ZFX-siRNA or scrambled siRNA and incubated at 37 °C for 4 days. After collection and washing with phosphate-buffered saline (PBS) buffer, cells were resuspended with staining buffer at a final density of 1 × 10^6^–1 × 10^7^/ml. Then, 5 μl annexin V-APC was added to 100 μl cell suspensions and incubated at room temperature in the dark for 10–15 min. Finally, cells were analyzed with FACS Calibur (Becton-Dickinson, USA) to determine cell apoptosis profiles.

### RNA extraction, reverse-transcription, and real-time quantitative PCR

To determine the siRNA efficiency, Tca-8113 cells were infected with lentivirus expressing either ZFX-siRNA or scrambled siRNA and cultured for another 48 h. Then, total RNA was isolated using Trizol reagent (Invitrogen) according to the manufacturer’s instructions. M-MLV reverse transcriptase (Promega) and Oligo dT primers (Sangon, Shanghai) were used for reverse transcription to produce cDNAs. Briefly, a 10 μl mixture containing 2 μg RNA, 0.5 μg Oligo dT primers was incubated at 70 °C for 10 min and then cooled on ice. Then, buffer, reverse transcriptase, RNase inhibitor, and dNTPs were added to a final 20 μl mixture and incubated at 42 °C for 1 h. ZFX expression was quantified with real-time quantitative PCR using a Real-Time PCR machine TP800 (Takara). Here, glyceraldehyde-3-phosphate dehydrogenase (GAPDH) was used as an internal control. Primers used in real-time PCR were as follows: GAPDH for 5′-TGACTTCAACAGCGACACCCA-3′; GAPDH reverse: 5′-CACCCTGTTGCTGTAGCCAAA-3′; ZFX for 5′-GGCAGTCCACAGCAAGAAC-3′; ZFX reverse: 5′-TTGGTATCCGAGAAAGTCAGAAG-3′. Messenger RNA (mRNA) levels of ZFX were normalized against GAPDH, and the comparative C_T_ method was chosen to quantify ZFX expression in Microsoft Excel.

### Colony formation assay

In brief, lentivirus expressing either ZFX-siRNA or scrambled siRNA was added into Tca-8113 cells. After incubation for another 48 h, cells reached the logarithmic phase and were harvested. Then, cell density was calculated using a hemocytometer, and 800 cells per well were plated in triplicate into 6-well plates. After culturing in a 5 % CO_2_ incubator at 37 °C for another 14 days, cells were fixed with paraformaldehyde for 30–60 min and then stained with GIEMSA for 20 min. Micropublisher 3.3RTV (Olympus) was used for imaging capture, and then, cell colonies under each condition were counted and analyzed.

### Cell cycle analysis by flow cytometry

Cell cycle process was analyzed using flow cytometry as described in previous studies with slight modifications [11]. In brief, lentivirus expressing either ZFX-specific siRNA or scrambled siRNA was added into human tongue squamous cell carcinoma cell line Tca-8113. After incubation for another 4 days, cells were resuspended and seeded in 6-cm dishes. Cells were collected and fixed with ice-cold 70 % alcohol after reaching approximately 80 % coverage. 40 × PI stock (2 mg/ml), 100 × RNase stock (10 mg/ml), and 1 × PBS buffer at a dilution of 25:10:1000 were used for subsequent cell staining. Then, flow cytometry machine FACS Calibur (Becton-Dickinson, USA) was used to perform cell cycle analysis. At least 1 × 10^6^ cells per sample were used for cell cycle analysis, and experiments were replicated for three times independently.

#### Western blot

In brief, Tca-8113 cells were washed with TBST buffer and then treated with lysis buffer. Appropriate protein lysates were mixed with 2× loading buffer, separated on 10–12 % SDS-PAGE and transferred to PVDF membranes (Amersham), which was further blocked with 5 % dissolved milk buffer for 2 h at room temperature and incubated overnight with specific primary antibodies including anti-cleaved caspase 3 (Cell Signaling #9664), anti-cleaved PARP (Cell Signaling #5625), and anti-GAPDH. After washing three times with TBST buffer, corresponding secondary antibodies coupled to HRP from Santa Cruz were added and ECL-Plus kit (Amersham Biosciences) was used for signal detection.

## Results

### Increased ZFX expression in tumors of tongue SCC patients

**Fig. 1:**
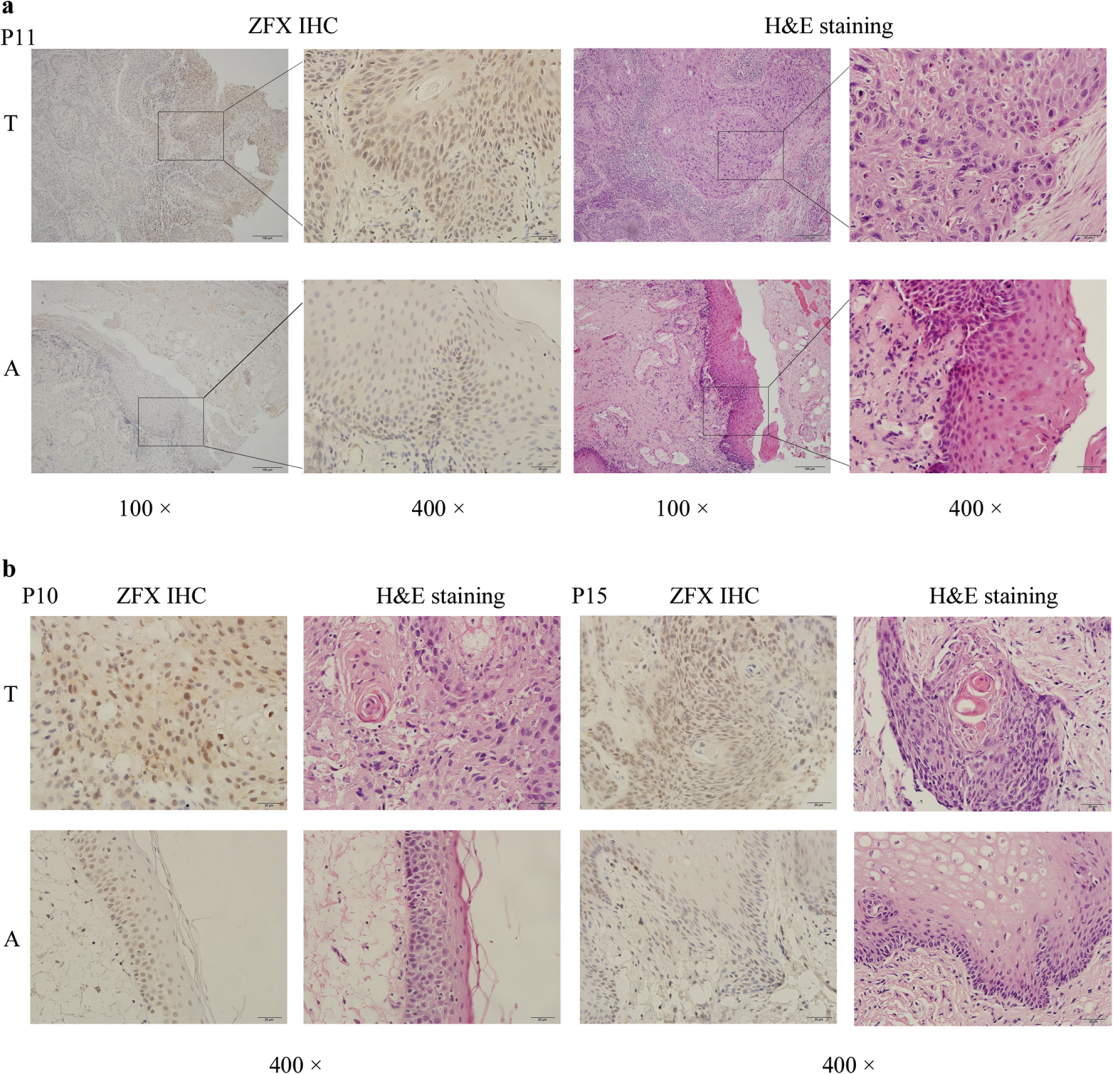
Expression of ZFX proteins in tumors and adjacent normal tissues from tongue SCC patients using IHC immunohistochemistry (IHC) and H&E staining. *T* tumor tissues, *A* adjacent normal tissues, *P11* patient 11, *P10* patient 10, *P15* patient 15. **a** Sample micrographs of ZFX IHC and H&E staining in tongue SCC tumor tissues and adjacent normal tissues from patient 11 (*left*, 100×, *right*, 400×). **b** Sample micrographs of ZFX staining and H&E staining in tongue SCC tumor tissues and adjacent normal tissues from patient 10 and patient 15 (*left*, patient 10, *right*, patient 15; magnification, 400×)

**Fig. 2:**
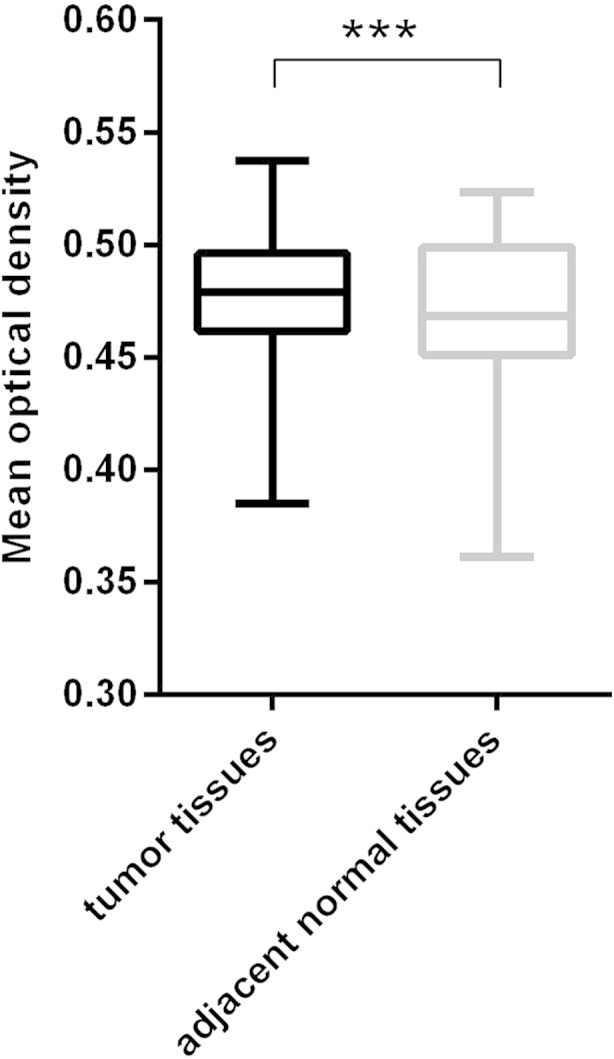
Expression quantification of ZFX proteins in tumor and adjacent normal tissues. Quantification of ZFX expression in tumors and tumor-adjacent normal tissues depending on optical density of ZFX positive signals in IHC slices

Initially, tumor tissues and adjacent tumor tissues collected from tongue SCC patients were examined and confirmed using hematoxylin and eosin (H&E) staining (representative images, Fig. 1a–b). Then, ZFX expression status was examined using immunohistochemistry (IHC) staining in tumor and adjacent normal tissues (representative images, Fig. 1a–b). ZFX expression were further quantified depending on the optical density of IHC signals and compared between tumors and adjacent normal tissues. Results showed that ZFX expression in tumor tissues was significantly higher than that in adjacent normal tissues (Fig. 2).

### ZFX expression was suppressed using lentiviral-mediated siRNA strategy in Tca-8113 cell lines

**Fig. 3:**
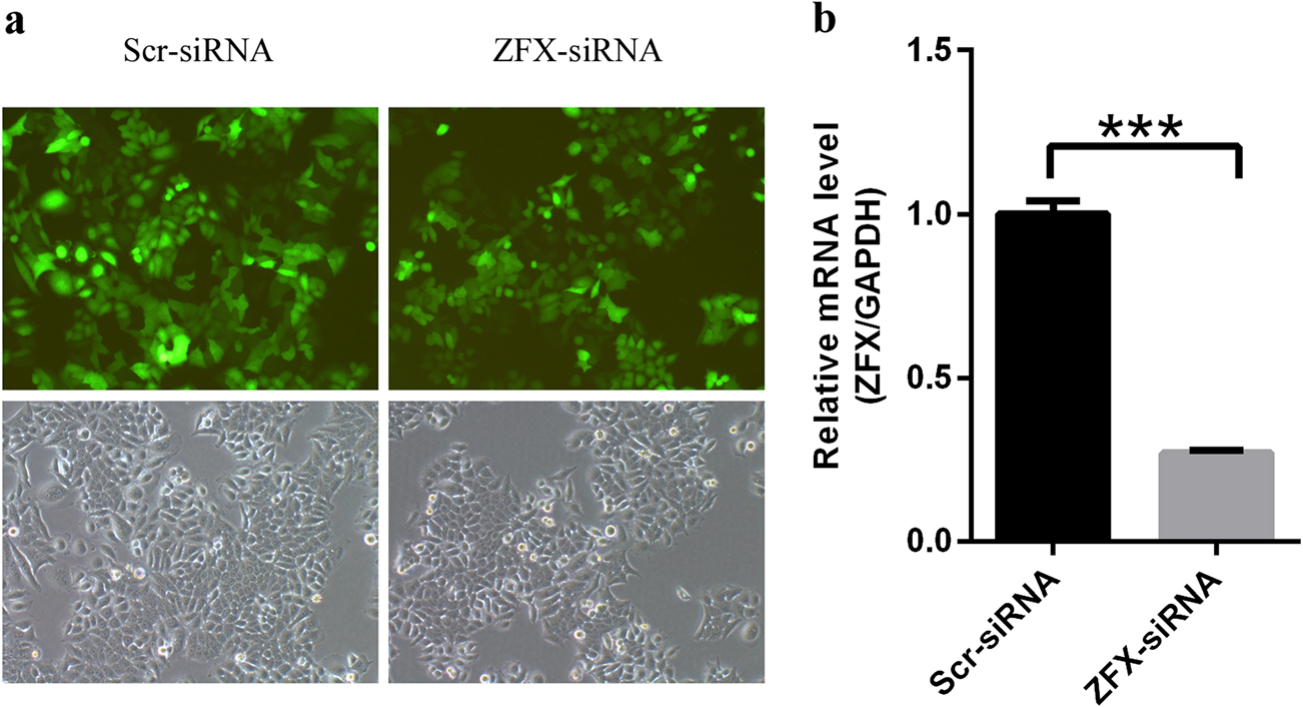
ZFX knockdown by lentiviral-based siRNA strategy. **a** Representative pictures of Tca-8113 cells infected with lentivirus expressing either scr-siRNA or ZFX-siRNA for 48 h. **b** ZFX expression at mRNA level in cells infected with lentivirus expressing either scr-siRNA or ZFX-siRNA was analyzed using real-time quantitative PCR method. GAPDH was used as internal control, and data shown here is the mean ± S.D. of three independent experiments (***p < 0.001)

As described above, ZFX expression in human tongue SCC samples was significantly higher than that in normal adjacent tissues, which implicated that ZFX might be a pathological factor involved in human tongue SCC development. Tca-8113 cell line from human tongue squamous cell carcinoma was selected for ZFX functional analysis in human tongue SCC development in vitro. And, lentiviral-mediated small interfering RNA (siRNA) strategy was employed to inhibit ZFX expression in Tca-8113 cells. Lentivirus expressing either scr-siRNA or ZFX-siRNA was generated and added into Tca-8113 cells. GFP expression could be observed in more than 95 % of cells 48 h after lentivirus tranfection (Fig. 3a). After 48 h incubation, total RNAs were extracted from cells infected with lentivirus expressing scr-siRNA or ZFX-siRNA and ZFX expression status at mRNA level was determined using real-time quantitative PCR. It was shown that ZFX expression in cells infected with ZFX-siRNA was downregulated by 73 % as compared to cells infected with scr-siRNA (Fig. 3b). Our results provided strong evidence showing that lentiviral-mediated siRNA strategy could inhibit ZFX expression efficiently in Tca-8113 cells, which provided a reliable method for ZFX functional analysis in subsequent experiments.

### ZFX knockdown inhibited cell proliferation inTca-8113 cells

**Fig. 4:**
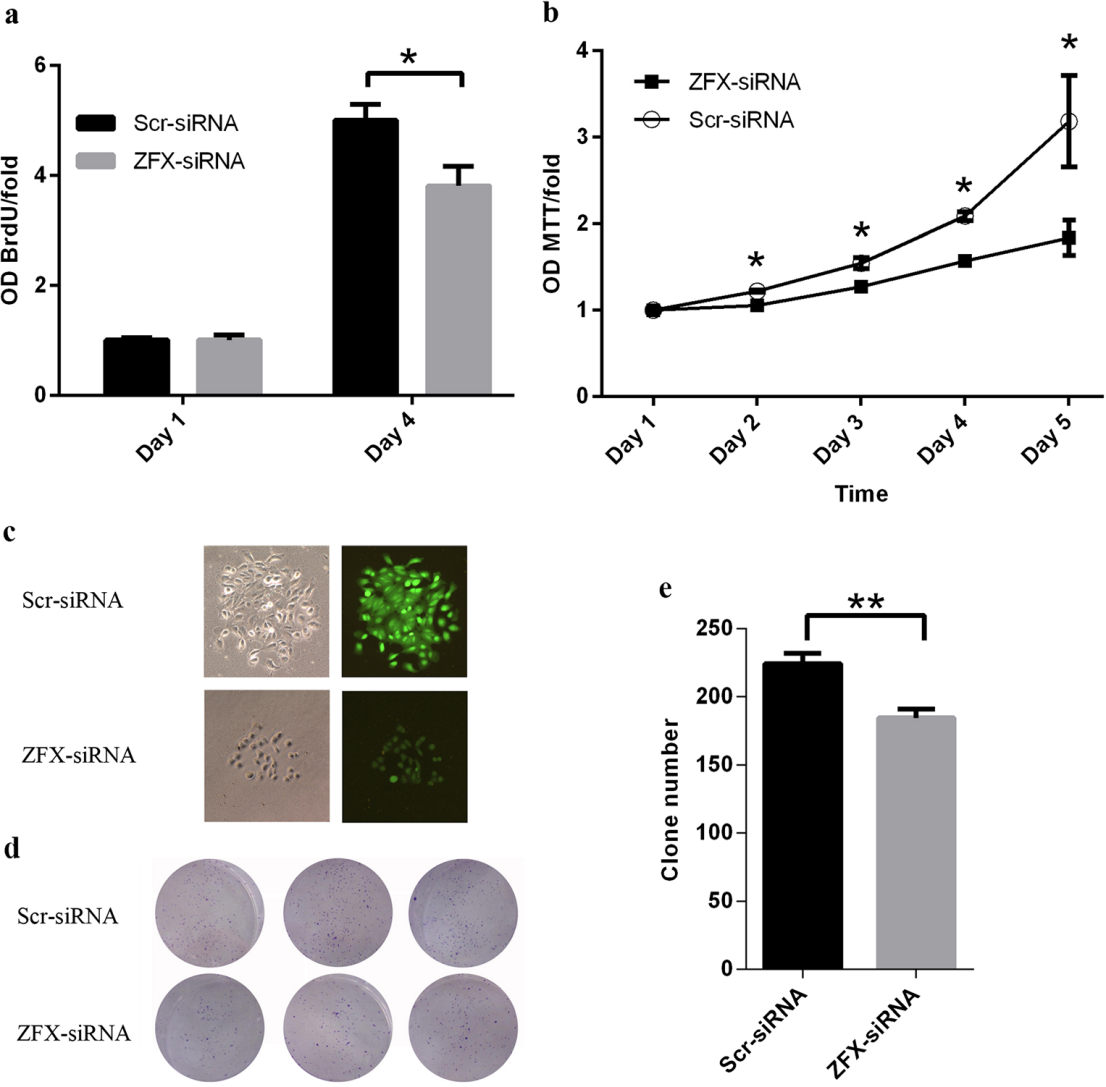
Impairment of cell proliferation and colony formation ability in Tca-8113 cells infected with lentivirus expressing ZFX-siRNA. **a** BrdU incorporation assay was used to analyze the proliferation of Tca-8113 cells infected with lentivirus expressing either scr-siRNA or ZFX-siRNA. The BrdU incorporation ratio is represented as fold changes of absorbance at 450 nm (OD_BrdU_/fold). Day 1 means the BrdU incorporation ratio in cells 24 h after lentivirus infection, and day 4 means the BrdU incorporation ratio 4 days after lentivirus infection. Data here is the mean ± S.D. of three independent experiments (*p < 0.05). **b** Cell proliferation of Tca-8113 cells infected with lentivirus expressing either scr-siRNA or ZFX-siRNA was further determined by MTT assay for continuous 5 days. Data shown here is the mean ± S.D. from three independent experiments (*p < 0.05). **c** Representative pictures of clones formed by Tca-8113 cells infected with lentivirus expressing either scr-siRNA or ZFX-siRNA. Differential interference contrast (DIC, *left panel*) and GFP (*right panel*) pictures were shown. **d** Representative pictures of colony formation assay in 6-well plate for Tca-8113 cells infected by lentivirus expressing either scr-siRNA or ZFX-siRNA. **e** Quantification of clone number in Tca-8113 cells infected by lentivirus expressing either scr-siRNA or ZFX-siRNA. Data shown here is the mean ± S.D. of clone numbers from three independent experiments (**p < 0.01)

Sustained proliferation ability is the most fundamental hallmark of cancer cells. So, here, Tca-8113 cells were treated with lentivirus expressing ZFX- or scr-siRNA, and BrdU incorporation assay was employed to investigate the impact of ZFX knockdown on cell proliferation ability. BrdU incorporation ratio was determined after 24 h of cell seeding, and it was shown that initial proliferation ability of Tca-8113 cells with different treatment was comparable (Fig. 4a, day 1). However, after 4 days of lentivirus infection, ZFX-specific siRNA resulted in remarkably impaired cell proliferation with about 24 % reduction of BrdU incorporation ratio (Fig. 4a, day 4). Cell proliferation status was further analyzed with MTT assay for continuous 5 days. At the first day, cell proliferation was comparable in Tca-8113 cells treated with either scr-siRNA or ZFX-siRNA. However, in the following 4 days, cell proliferation was significantly impaired in cells treated with ZFX-siRNA as compared to cells treated with scr-siRNA (Fig. 4b). Our results revealed that when ZFX was inhibited, Tca-8113 cell proliferation was suppressed significantly.

### Impairment of colony formation ability in Tca-8113 cells by ZFX knockdown

Clonogenic ability is critical for tumor outgrowth and expansion and could be determined using colony formation assay, which examines the ability of a single cell to form a colony containing at least 50 cells. And here, it was shown that smaller clones containing sparse and fewer cells were formed by Tca-8113 cells targeted by ZFX-siRNA as compared to clones formed by Tca-8113 cells targeted by scr-siRNAs (Fig. 4c, left panel). Furthermore, fluorescence in clones formed by ZFX-siRNA-treated cells was significantly weaker than that in clones formed by scr-siRNA-treated cells (Fig. 4c, right panel). And, colony formation assay showed that an average of 224 clones formed in scr-siRNA-treated cells while the clone number was reduced to 185 in ZFX-siRNA-treated cells (Fig. 4d–e). These results revealed that ZFX knockdown inhibited clone formation in Tca-8113 cells, which indicated that ZFX is essential for clonogenic ability of human tongue squamous cell carcinoma cells.

### Cell cycle disruption by ZFX knockdown

**Fig. 5:**
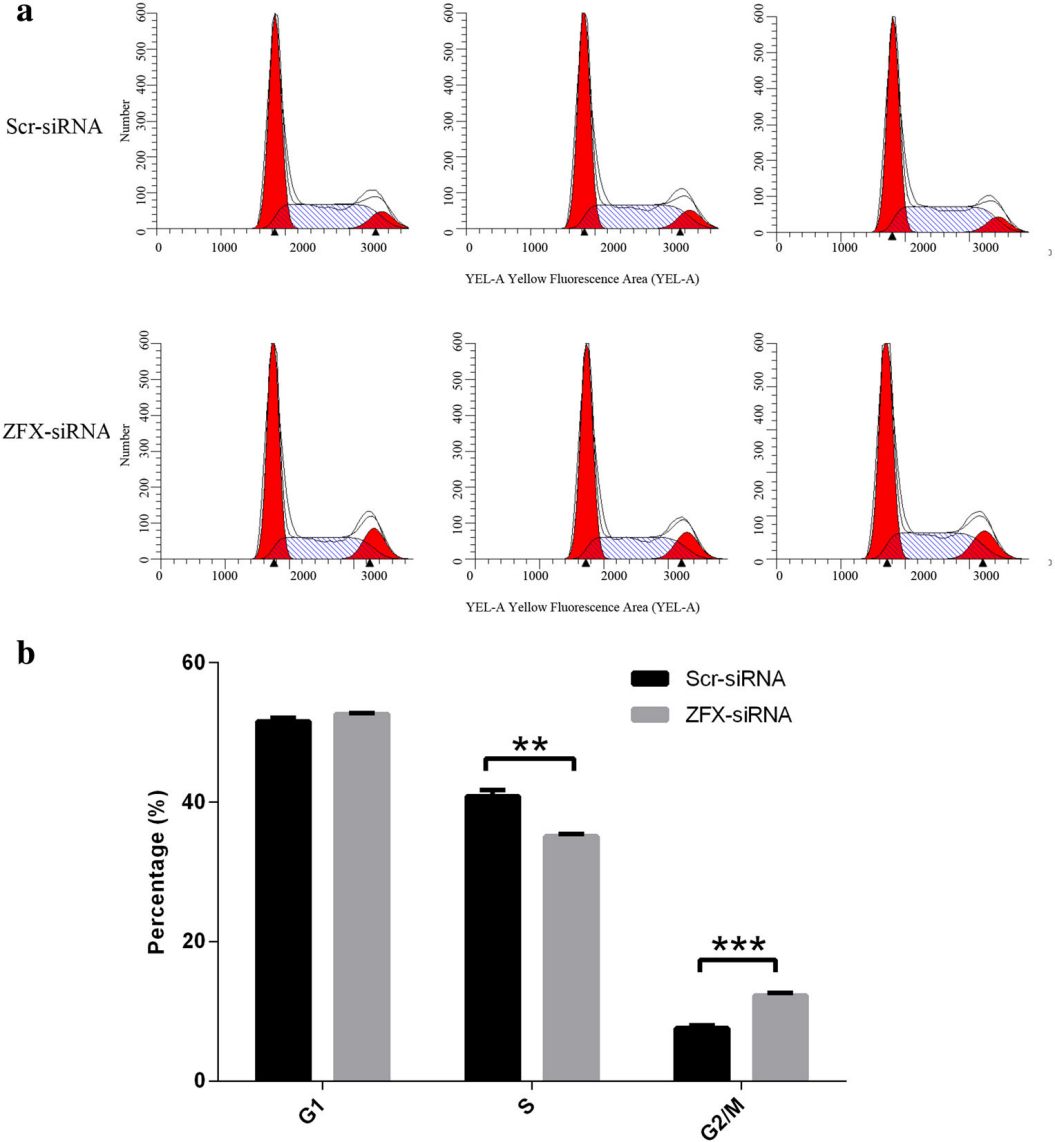
Cell cycle disruption in Tca-8113 cells infected with lentivirus expressing ZFX-specific siRNA. **a** Cell cycle analysis by flow cytometry and representative images were shown. **b** Cell cycle distribution in Tca-8113 cells infected with lentivirus expressing scr-siRNA or ZFX-siRNA. Data shown here is the mean ± S.D. of cell percentage from three independent experiments (**p < 0.01, ***p < 0.001)

Impairment of cell proliferation by ZFX knockdown promoted us to examine the relationship between ZFX and cell cycle progression in Tca-8113 cells. Cells were treated with lentivirus expressing either scr-siRNA or ZFX-siRNA, and cell cycle was analyzed with flow cytometry (Fig. 5a). Results showed that significant cell cycle arrest at the G2/M phase was induced by ZFX knockdown, as the percentage of G2/M phase cells increased from 7.59 to 12.27 % with ZFX-siRNA treatment. In addition, cell percentage at S phage was significantly decreased from 40.85 to 35.08 %, but cell percentage at G1 phase changed little (Fig. 5b). Taken together, these data showed that cell cycle progression was disrupted by ZFX knockdown and indicated that ZFX knockdown-induced cell proliferation impairment might be partially attributed to cell cycle arrest.

### Reduction of ZFX induced apoptosis in Tca-8113 cells

**Fig. 6:**
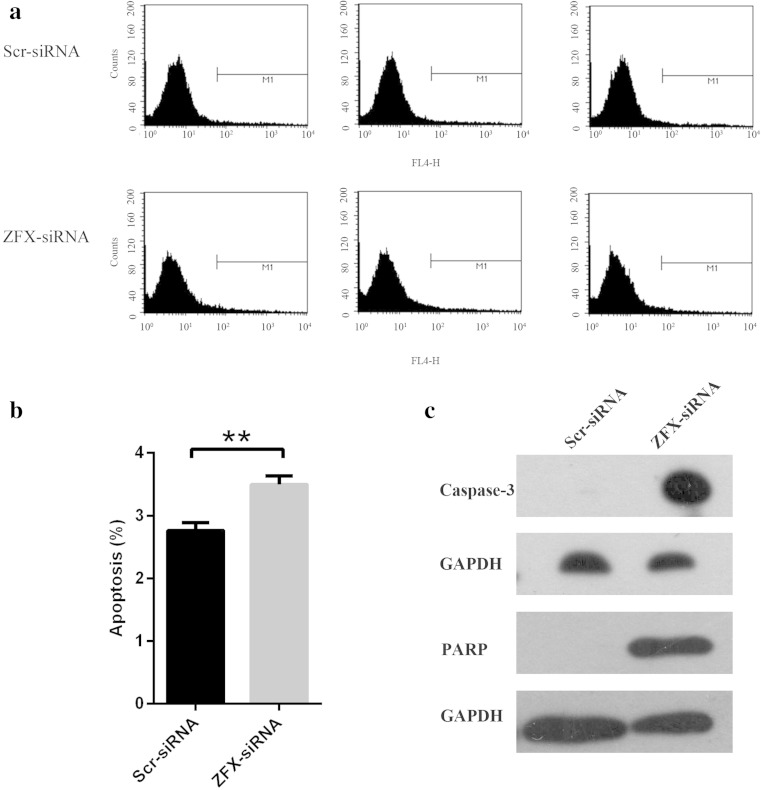
Apoptosis induction in Tca-8113 cells infected with lentivirus expressing ZFX-specific siRNA. **a** Cell apoptosis by annexin-V staining was analyzed with flow cytometry. **b** Cell apoptosis in Tca-8113 cells infected with lentivirus expressing scr-siRNA or ZFX-siRNA. Apoptosis was determined with annexin V-APC assay and analyzed using flow cytometry. Data shown here is the mean ± S.D. of cell percentage in apoptosis from three independent experiments (**p < 0.01). **c** Western blot analysis of apoptosis-associated genes in Tca-8113 cell lysates infected with lentivirus expressing scr-siRNA or ZFX-siRNA. GAPDH was used as internal control

In addition to uncontrolled proliferation, apoptosis evasion is another fundamental trait of cancer cells. To examine the impact of ZFX knockdown on cell apoptosis, Tca-8113 cells were infected with lentivirus expressing ZFX-specific or scrambled siRNA. After 4 days of lentivirus infection, the apoptosis of cells was determined with annexin V-APC assay and analyzed by flow cytometry (Fig. 6a). Apoptosis was observed in 2.76 % of Tca-8113 cells infected with scrambled siRNA and increased to 3.5 % in cells infected with ZFX-specific siRNA (Fig. 6b). Furthermore, Western blot analysis was performed to determine whether any apoptosis-associated genes were involved in the process of ZFX-induced cell apoptosis in Tca-8113 cells. It was shown that ZFX knockdown led to the upregulation of cleaved caspase 3 and PARP proteins (Fig. 6c), which were both critical for the induction of cell apoptosis. Taken together, these data suggests that ZFX plays an anti-apoptotic role in Tca-8113 cells.

## Discussion

ZFX protein is a C2H2-ZF protein identified as a transcriptional factor belonging to ZFY protein family. Its involvement in cancer development has been explored extensively. For example, ZFX overexpression has been observed in prostate cancer [12], gastric cancer [13–15], human gliomas [16, 17], non-small-cell lung cancer (NSCLC) [18], breast cancer [19], and so on. Studies from our lab also showed that ZFX is involved in human laryngeal squamous cell carcinoma (LSCC), a subgroup of cancer of HNSCC, and ZFX knockdown impaired the cell proliferation and induced apoptosis in LSCC Hep-2 cells [7]. Here, we unraveled that ZFX expression increased significantly in tongue SCC tumors, another type of cancer of HNSCC, as compared to tumor-adjacent normal tissues. So, these results suggest that ZFX upregulation in tumors is quite common and ZFX as a potential biomarker for cancer diagnosis should be investigated in the future.

Tongue SCC accounts for about 90 % of HNSCC, which ranks among the top ten cancers worldwide. However, disrupted molecular factors underlying tongue SCC initiation and progression remain poorly understood, which hindered the development of efficient strategies for tongue SCC prevention, diagnosis, prognosis, and treatment. Here, we quantified ZFX expression in tissue samples from tongue SCC patients by OD measurement rather than by pathologists, as OD measurement will provide objective quantifications about ZFX expression. And, our discovery of ZFX overexpression in tongue SCC samples expanded our understanding about molecular mechanisms of tongue SCC. Furthermore, in vitro studies in Tca-8113 cells revealed that ZFX inhibition by lentivirus-based siRNA strategy delayed cell proliferation, impaired colony formation ability, and lead to cell cycle arrest while promoted cell apoptosis, suggesting its essential role for tumor cell growth and survival. However, the underlying mechanisms about colony formation ability impairment by ZFX knockdown remain elusive, though it was shown in this study that ZFX expression is essential for colony formation. In-depth analysis of ZFX-associated molecular pathways will provide more valuable insights in tongue SCC and HNSCC development.

Indeed, it has been reported that ZFX is the target gene of miR-144, and in consistent with the oncogenic functions of ZFX, miR-144 expression was diminished significantly in gastric cancer and non-small-cell lung cancer [14, 18], suggesting that disrupted miR-144-ZFX axis may be underlying the development of variant tumors. Studies also showed that miR-144 overexpression could inhibit the tumor growth and induce cell apoptosis in NSCLC which is in accordance with the effect of ZFX knockdown. In addition to the upstream effector miR-144, downstream effectors of ZFX were also investigated in other studies. It has been reported that ZFX knockdown in diverse tumor types including gliomas [17], NSCLC [20], LSCC [7], breast cancers [19], and gastric cancer [13] all led to hypophosphorylation of Akt, extracellular signal-regulated kinase 1/2 (ERK1/2), or MEK1/2 except for ERK2 hyperphosphorylation in breast cancer cells [19]. These results indicated that ZFX was critical for the activation of Akt and MAPK signaling pathways, which were two important pathways for cell survival. What’s more, ZFX knockdown resulted in the downregulation of anti-apoptotic factor such as bcl-2 and upregulation of apoptotic factors including bax, caspase1, 3, 9 in diverse cancers [7, 12, 13, 17, 19, 20]. In our study, we also confirmed that two apoptosis-related proteins cleaved caspase 3 and PARP were both upregulated in Tca-8113 cells treated with ZFX-specific siRNA. These results were all in consistent with the phenomenon that ZFX knockdown induces cell apoptosis in Tca-8113 cells and provided valuable insights for the underlying mechanisms of ZFX-mediated cell apoptosis. Furthermore, it has been revealed that cell cyle factors such as cyclin D1 and cyclin B1 was downregulated by ZFX knockdown in multiple cancer types [17, 19, 20], which might account for the cell cycle disruption in Tca-8113 cells treated with ZFX-siRNA. Indeed, ZFX knockdown induced G1 phase cell cycle arrest in Tca-8113 cells, which is in consistent with previous cell cycle results in PC-3 cells [12], SGC7901 cells [13], H1299 cells [20], MDA-MB-231 cells [19], U87, and U373 cells [21], and in contrast to results in MGC803 cells [13] and Hep-2 cells [7], in which S phase cell cycle arrest was induced by ZFX knockdown. It has been demonstrated that cyclin D1 and cyclin E1 were essential for G1/S phase transition [22, 23] while cyclin A2 was involved in S-phase process [24], so it is quite likely that cyclin E1 or cyclin D1 function was disrupted in Tca-8113 cells by ZFX knockdown, which has been confirmed in SGC7901 cells [13], U87, and U373 cells [17] treated with ZFX-siRNA. It should be noticed that though ZFX functions have been investigated in multiple cancers, very little research focused on the roles of ZFX in HNSCC or tongue squamous cell carcinoma. So, our future studies will focus on the upstream/downstream pathways of ZFX in vitro and in vivo to provide more insights into the underlying mechanisms about HNSCC or tongue squamous cell carcinoma. A thorough and comprehensive analysis about ZFX in different cancers are also needed, and such analysis will help to clarify the distinct and overlapping roles of ZFX in different cancers, paving the way for the development of efficient clinical applications targeting ZFX.
